# Does the Site of Origin of the Microcarcinoma with Respect to the Thyroid Surface Matter? A Multicenter Pathologic and Clinical Study for Risk Stratification

**DOI:** 10.3390/cancers12010246

**Published:** 2020-01-19

**Authors:** Giovanni Tallini, Antonio De Leo, Andrea Repaci, Dario de Biase, Maria Letizia Bacchi Reggiani, Doriana Di Nanni, Francesca Ambrosi, Cira Di Gioia, Giorgio Grani, Kerry Jane Rhoden, Erica Solaroli, Fabio Monari, Sebastiano Filetti, Cosimo Durante

**Affiliations:** 1 Department of Experimental, Diagnostic and Specialty Medicine, University of Bologna–Molecular Diagnostic Unit, 40138 Azienda USL di Bologna, Italy; d.dinanni@gmail.com (D.D.N.); fra.ambrosi@gmail.com (F.A.); 2 Pathology Unit, Department of Experimental, Diagnostic and Specialty Medicine, University of Bologna, S. Orsola-Malpighi Hospital, 40138 Bologna, Italy; antonio.deleo@unibo.it; 3 Endocrinology Unit, Department of Medical and Surgical Sciences, University of Bologna, S. Orsola-Malpighi Hospital, 40138 Bologna, Italy; rep.rep@libero.it; 4 Department of Pharmacy and Biotechnology, University of Bologna–Molecular Diagnostic Unit, Azienda USL di Bologna, University of Bologna, 40138 Bologna, Italy; dario.debiase@unibo.it; 5 Department of Experimental, Diagnostic and Specialty Medicine, University of Bologna, S. Orsola-Malpighi Hospital, 40138 Bologna, Italy; maria.bacchireggiani@unibo.it; 6 Department of Radiological, Oncological and Pathological Sciences, “Sapienza” University of Rome, 00185 Rome, Italy; cira.digioia@uniroma1.it; 7 Department of Translational and Precision Medicine, “Sapienza” University of Rome, 00185 Rome, Italy; giorgio.grani@uniroma1.it (G.G.); cosimo.durante@uniroma1.it (C.D.); 8 Genetics Unit, Department of Medical and Surgical Sciences, University of Bologna, S. Orsola-Malpighi Hospital, 40138 Bologna, Italy; kerry.rhoden@unibo.it; 9 Endocrinology Unit, Ospedale Maggiore, 40133 Bologna, Italy; e.solaroli@ausl.bologna.it; 10 Radiation Therapy Unit, S. Orsola-Malpighi Hospital, 40138 Bologna, Italy; fabio.monari@aosp.bo.it

**Keywords:** papillary microcarcinoma, thyroid nodule, thyroid capsule, tumor diameter, tumor origin, tumor location, risk stratification, papillary carcinoma prognosis

## Abstract

It is unclear whether the site of origin of papillary thyroid microcarcinoma (mPTC) with respect to the thyroid surface has an influence on clinicopathologic parameters. The objectives of the study were to: (i) Accurately measure the mPTC distance from the thyroid surface; (ii) analyze whether this distance correlates with relevant clinicopathologic parameters; and (iii) investigate the impact of the site of origin of the mPTC on risk stratification. Clinicopathologic features and *BRAF* mutational status were analyzed and correlated with the site of origin of the mPTC in a multicenter cohort of 298 mPTCs from six Italian medical institutions. Tumors arise at a median distance of 3.5 mm below the surface of the thyroid gland. Statistical analysis identified four distinct clusters. Group A, mPTC: size ≥ 5 mm and distance of the edge of the tumor from the thyroid capsule = 0 mm; group B, mPTC: size ≥ 5 mm and distance of the edge of the tumor from the thyroid capsule > 0 mm; group C, mPTC: size < 5 mm and distance of the edge of the tumor from the thyroid capsule = 0 mm; and group D, mPTC: size < 5 mm and distance of the edge of the tumor from the thyroid capsule > 0 mm. Univariate analysis demonstrates significant differences between the groups: Group A shows the most aggressive features, and group D the most indolent ones. By multivariate analysis, group A tumors are characterized by tall cell histotype, *BRAF* V600E mutation, tumor fibrosis, aggressive growth with invasive features, vascular invasion, lymph node metastases, and intermediate ATA risk. The mPTC clinicopathologic features vary according to the tumor size and distance from the thyroid surface. A four-group model may be useful for risk stratification and to refine the selection of nodules to be targeted for fine needle aspiration.

## 1. Introduction

In the recent past, papillary thyroid microcarcinoma (mPTC) has come under the spotlight as the main culprit of the so-called thyroid cancer “epidemic” [1], caused by overscreening and resulting in the overdiagnosis of small indolent tumors. The incidence of thyroid cancer has currently increased in many countries without an associated increase in morbidity or mortality. The increase has been primarily due to the widespread utilization of imaging techniques that allow the identification of a large number of small thyroid tumors, including mPTC (i.e., papillary carcinoma <or =10 mm) [1,2]. To avoid cancer overdiagnosis, categories, such as papillary microtumor (PMiT) [3] and noninvasive follicular thyroid neoplasm with papillary-like nuclear features (NIFTP), have been proposed for the diagnosis of thyroid neoplasms with indolent features, including mPTC [4,5].

In fact, small tumors of the thyroid gland with distinctive clinicopathologic features have long been recognized [6], as early as 1928 [7]. These tumors received different names, such as adenocarcinoma not originating in an adenoma [7], nonencapsulated sclerosing tumors of the thyroid [8], and occult sclerosing carcinoma [9]. The clear recognition that this tumor is a subtype of papillary carcinoma and the very term microcarcinoma are credited to Hazard in 1960 [10]. 

Since their original description, the features that characterize mPTC were the peripheral location within the gland, together with small size and discovery as an incidental finding [6]. Microcarcinomas have always been considered clinically indolent, but their potentially malignant behavior has also been recognized due to a rate of lymph node metastases that is not negligible, although the prognostic role of these lymph node metastases has probably been overestimated [11]. 

Although the peripheral location, close to the surface of the thyroid gland (the so-called thyroid capsule), was recognized in early studies, surprisingly, the distribution of the site of origin of mPTCs with respect to the surface of the gland has never been accurately measured, and the clinicopathologic implications of this distribution have never been fully addressed.

The aims of this study were to analyze: (i) The distance of the mPTC from the thyroid capsular surface; (ii) whether this distance correlates with relevant clinicopathologic parameters, including tumor subtype and *BRAF* V600E status, characteristics of tumor growth, and microscopic appearance of the tumor; and (iii) what is the potential impact of the site of origin of the mPTC with respect to the thyroid capsule on risk stratification.

## 2. Results

To understand whether the origin of the mPTC within the thyroid gland—in particular its subcapsular vs. nonsubcapsular location—correlates with clinicopathologic features, we analyzed a series of 298 mPTCs from 264 patients originating from different Italian regions. All cases were subjected to central review and meticulous histopathologic analysis.

**Figure 1:**
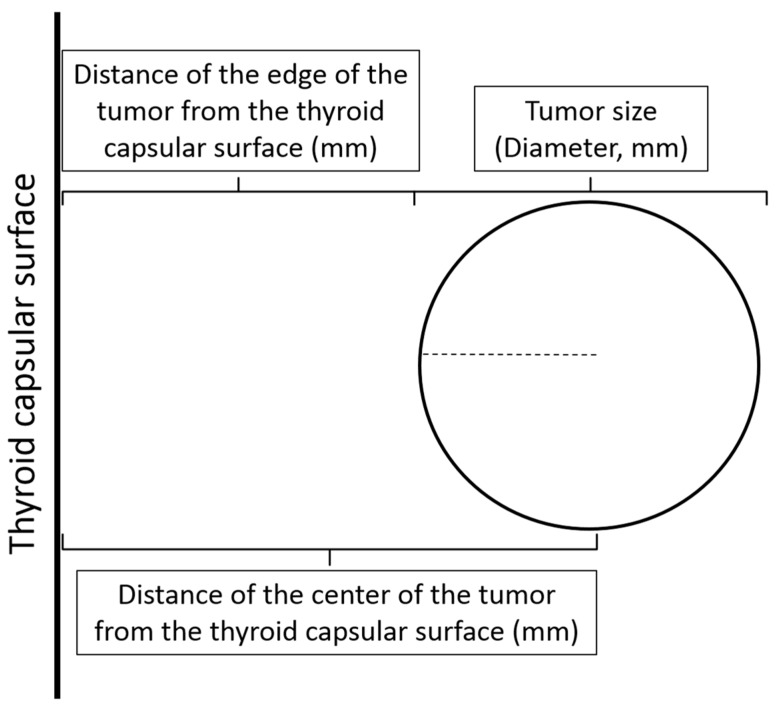
Distance of the center of the tumor from the thyroid surface. The distance of the center of the tumor from the thyroid surface is measured by adding the separate micrometric values of the distance of the edge of the tumor from the thyroid surface to the radius of the tumor (tumor diameter (mm)/2).

The distance of the center of the tumor from the thyroid capsule was calculated as shown in Figure 1 (see also Materials and Methods). The large majority, 286 of 298 (96%), of mPTCs reached within 5 mm of the thyroid capsule, irrespective of the overall dimensions of the thyroid gland or the mPTC diameter.

Since the distance of the edge of the tumor from the thyroid surface in 12 of the 298 tumors was greater than 5 mm, it could not be accurately calculated (or estimated) on histology sections. These 12 mPTCs were therefore excluded from the analysis. Statistical analysis was thus conducted on 286 mPTCs from 257 patients, 29 of whom with multicentric tumors.

**Figure 2:**
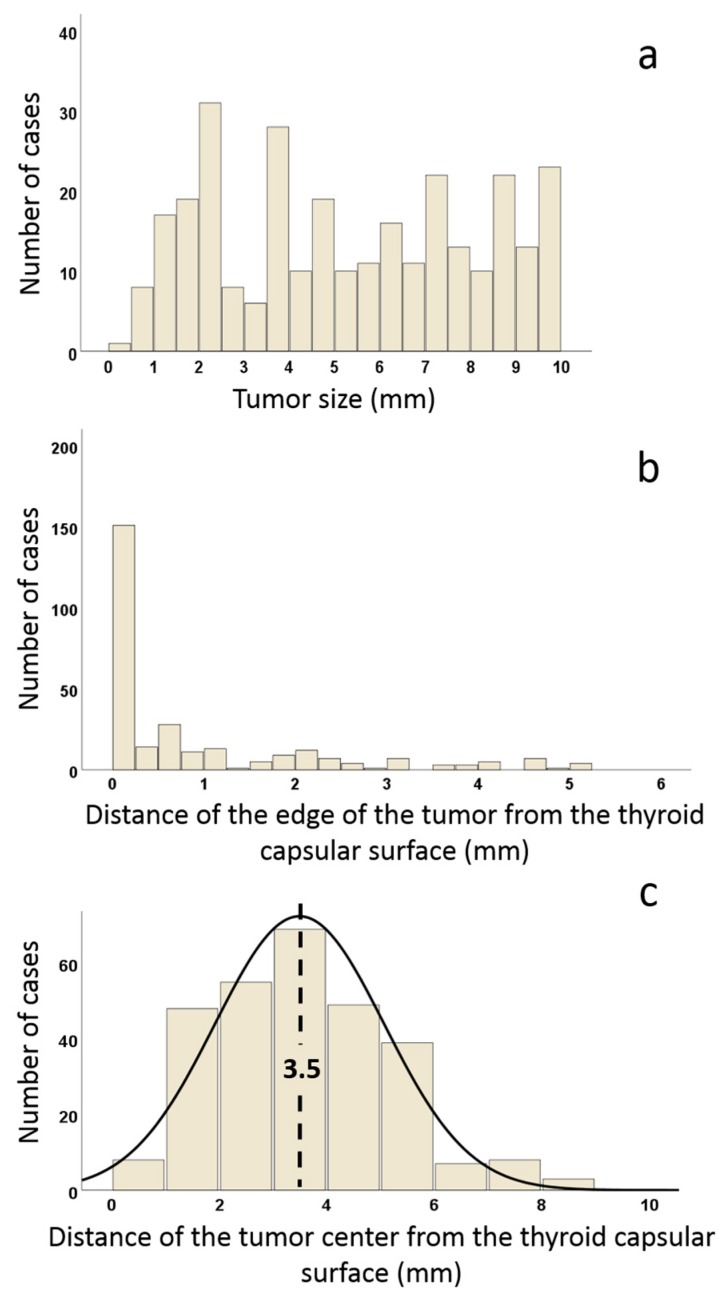
Distribution of cases according to tumor size (**a**), to the distance of the edge of the tumor from the thyroid capsule (**b**), and according to the distance of the tumor center from the thyroid capsule (**c**). The median distance of the tumor center from the thyroid capsule is 3.5 mm (range 0.5–11 mm).

Figure 2 shows the distribution of our cases according to the mPTC size (Figure 2a) and distance of the edge of the tumor from the thyroid capsule (Figure 2b). The distribution of mPTCs is random according to size (Figure 2a) but is skewed according to the distance of their edge from the thyroid capsule because the majority of mPTCs reach close to the thyroid surface (Figure 2b). Figure 2c shows a bell-shaped normal distribution of mPTCs according to the distance of the tumor center from the thyroid capsule, with a median distance of 3.5 mm (range 0.5–11 mm).

The clinicopathologic features of the 286 tumors are summarized in Table 1.

**Figure 3:**
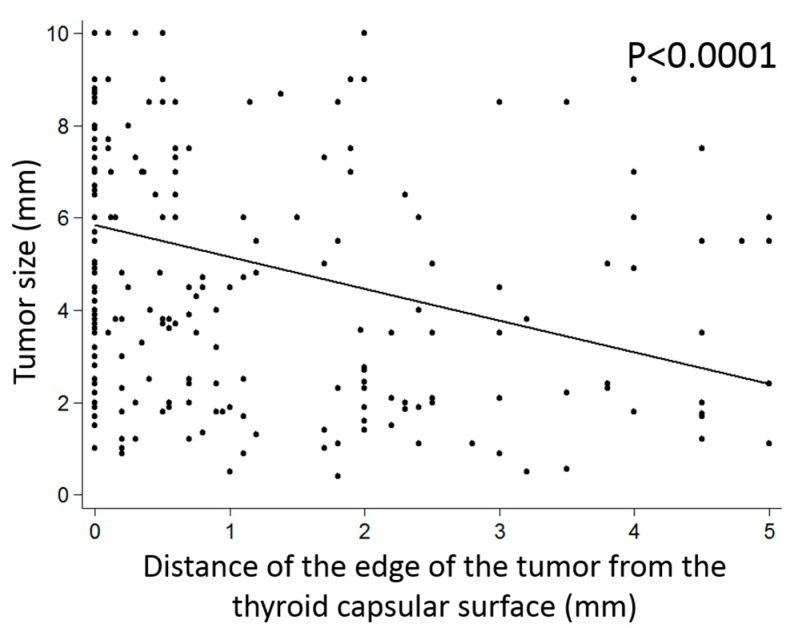
Relationship of the tumor size with the distance of the edge of the tumor from the thyroid capsule. Small microcarcinomas are preferentially located away from the capsule (linear regression, *p* < 0.0001).

First, we studied the relationship between the distance of the center of the tumor from the thyroid surface and the clinicopathologic variables (including tumor subtype and *BRAF* V600E status), the characteristics of tumor growth, and the microscopic appearance of the tumor. Univariate analysis highlighted significant correlations with important characteristics of the mPTCs (Table S1 and Supplementary Materials), apparently indicating that tumors with aggressive features arise further away from the thyroid capsule. However, the results were heavily influenced and biased by the tumor size: Larger tumors are by definition centered further away from the thyroid capsule when compared with mPTC of a smaller size. In addition, statistical analysis (linear regression, *p* < 0.0001) shows that the tumor size and the distance of the edge of the tumor from the thyroid capsular surface are not independent variables. In fact, small mPTCs are preferentially located away from the thyroid surface: The smaller the tumor size, the further the distance of the edge of the tumor from the thyroid surface (*p* < 0.0001) (Figure 3). Thus, the distribution of mPTCs according to their size and to the distance of their edge from the thyroid capsular surface is clearly uneven, with larger tumors preferentially located closer to and smaller ones further away from the thyroid surface (Figure S1).

We therefore postulated four mPTC groups based on the tumor size and the relationship of the tumor with the thyroid surface as follows: 

Group A: Tumors with size ≥5 mm and distance of the edge of the tumor from the thyroid surface = 0 mm (large subcapsular mPTC) 

Group B: Tumors with size ≥5 mm and distance of the edge of the tumor from the thyroid surface >0 mm (large nonsubcapsular mPTC) 

Group C: Tumors with size <5 mm and distance of the edge of the tumor from the thyroid surface =0 mm (small subcapsular mPTC) 

Group D: Tumors with size <5 mm and distance of the edge of the tumor from the thyroid surface >0 mm (small nonsubcapsular mPTC) 

**Figure 4:**
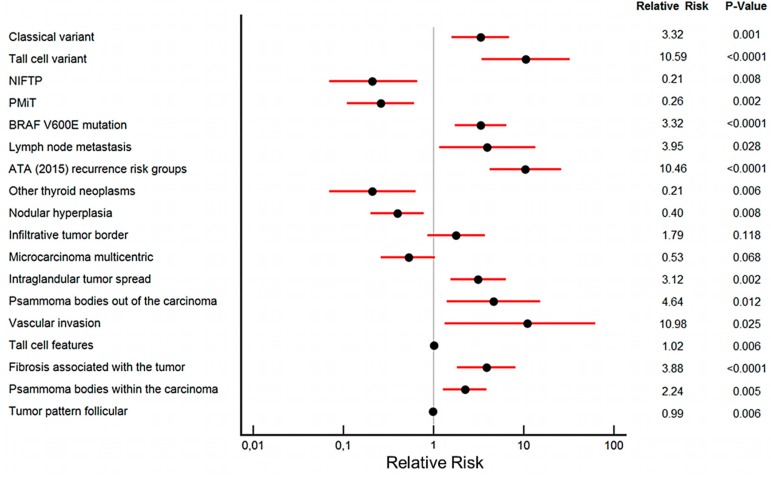
Multinomial logistic regression analysis comparing the four microcarcinoma groups (group D tumors used as a reference).

The distribution of the four groups according to the distance of the tumor center from the thyroid surface is shown in Figure S2. The correlation of these four mPTC groups with clinicopathologic features, including tumor subtype and *BRAF* V600E status, the characteristics of tumor growth, and the microscopic appearance of the tumor, was analyzed to investigate their possible relevance for risk stratification (Table 2, Figure 4, Table S2a,b). Univariate analysis demonstrates significant differences between the four groups, with group A showing the most aggressive features and group D the most indolent ones (see Table 2). Important features statistically linked to group A include: Tall cell histotype (*p* < 0.0001), *BRAF* V600E mutation (*p* < 0.0001), lymph node metastases (*p* = 0.003), and the targeting of the tumor for fine needle aspiration because of its worrisome ultrasound features (i.e., the mPTC was not an incidental finding in thyroid resections performed for other reasons, *p* = 0.006). Group A tumors are more frequently of the intermediate as opposed to low ATA risk group [12] and have a higher proportion of unfavorable disease-related patient events—persistent or recurrent disease (although this did not reach statistical significance due to the small number of cases with persistent or recurrent disease in our series). Group A tumors more commonly associate with invasion of extrathyroidal tissue (*p* < 0.0001), high-grade features (*p* = 0.029), mitoses (*p* = 0.049), and intra- and peritumoral lymphoid cell infiltration (*p* = 0.003 and *p* < 0.0001, respectively). Microscopically, group A tumors present distinctive features, such as a larger number of pseudoinclusions (*p* = 0.003), nuclear grooves (*p* = 0.004), and nuclear membrane irregularities (*p* = 0.0001); and a greater proportion of tumor cells with eosinophilic cytoplasm (*p* = 0.012), tall cell features (*p* = 0.0001), and papillary or with solid/trabecular pattern of growth (*p* = 0.0003 and *p* = 0.039, respectively). Very few of the group A cases can be subclassified as noninvasive follicular thyroid neoplasm with papillary-like nuclear features (NIFTP) (4 of 50 cases, 8.0%; *p* < 0.0001) [4,5] or papillary microtumor (PMiT) (10 of 69 cases, 14.5%; *p* < 0.0001) [3]. Conversely, group D tumors represent the large majority of all mPTCs subclassified as NIFTP (30 of 50 cases, 60%; *p* < 0.0001) or PMiT (40 of 69 cases, 58%; *p* < 0.0001). 

We subsequently performed a comprehensive multivariate analysis, including all the variables (clinicopathologic, tumor growth patterns, microscopic appearance) reported in Table 2, with *p* < 0.2 in the univariate analysis, to further define the clinical relevance for risk stratification of our four mPTC groups. The results are shown in Figure 4, and Table S2a,b.

Figure 4 summarizes the multivariate analysis using the most indolent mPTC group (group D tumors) as the reference for statistical analysis. Table S2b summarizes the multivariate analysis using the most aggressive mPTC group (group A tumors) as the statistical reference. 

**Figure 5:**
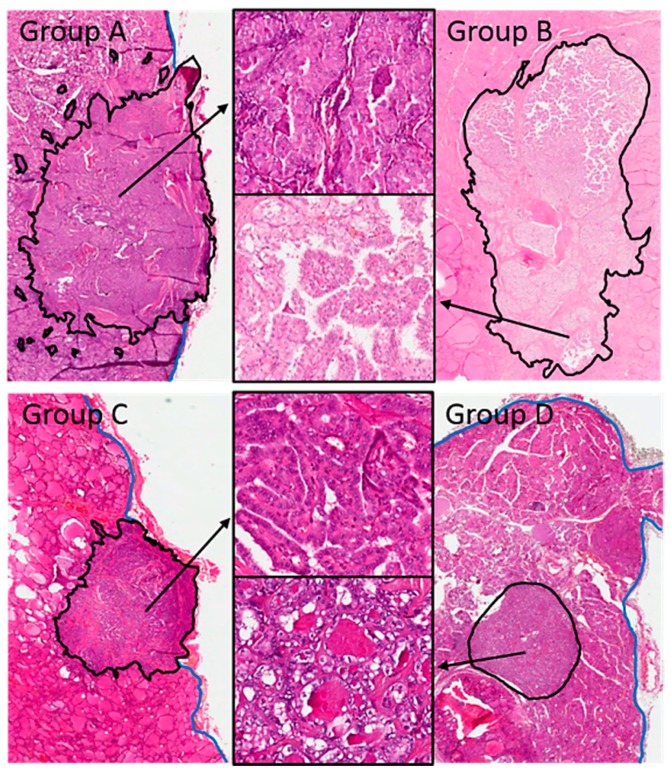
Histologic appearance of the microcarcinoma groups (hematoxylin and eosin sections). Group A (large subcapsular mPTC: size > or =5 mm and distance of the edge of the tumor from the thyroid capsule = 0 mm), group B (large nonsubcapsular mPTC: size > or =5 mm and distance of the edge of the tumor from the thyroid capsule >0 mm), group C (small subcapsular mPTC: size <5 mm and distance of the edge of the tumor from the thyroid capsule = 0 mm), and group D (small nonsubcapsular mPTC: size <5 mm and distance of the edge of the tumor from the thyroid capsule >0 mm). Low power appearance and histologic features at higher magnification (insets). The black lines outline the tumor border with the surrounding non-neoplastic tissue; the blue lines outline the capsular surface of the thyroid gland. Group A tumors represent the group with the most worrisome characteristics, and group D tumors that with the most indolent features.

**Figure 6:**
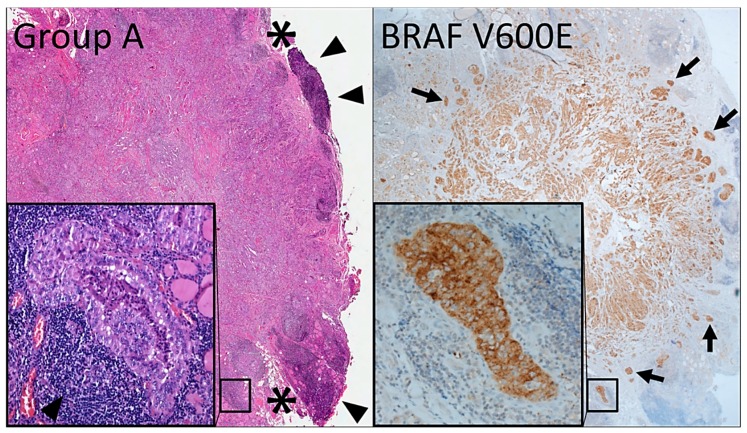
Histologic appearance (hematoxylin and eosin section, H&E) of group A microcarcinomas (left image and inset). This subcapsular tumor measures 9 mm, shows extrathyroidal extension (asterisks), is associated with conspicuous periperitumoral lymphoid cell infiltration (triangles), is of the tall cell histotype (left inset), and carries the *BRAF* V600E mutation. A serial section of the H&E is immunostained with a *BRAF* V600E-specific antibody (clone VE1) (right image and inset) to highlight the infiltrative nature of the tumor and intraglandular tumor spread (neoplastic cell aggregates separated from the tumor mass by non-neoplastic thyroid; inset and arrows).

**Figure 7:**
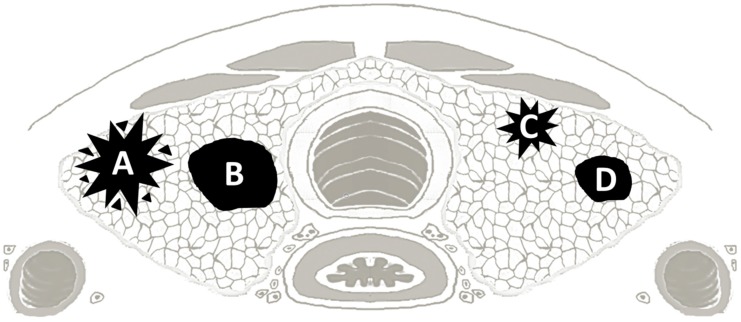
Schematic representation of the microcarcinoma groups A, B, C, and D.

Group A tumors independently correlate with specific clinicopathologic features: Tall cell or classic papillary carcinoma histotype (Table S2a; *p* < 0.0001 and *p* = 0.001, respectively), *BRAF* V600E mutation (Table S2a; *p* < 0.0001), lymph node metastases (Table S2a; *p* = 0.028), and intermediate as opposed to low American Thyroid Association (ATA) risk group (Table S2a; *p* < 0.0001). Group A mPTC are inversely related to NIFTP (Table S2a; *p* = 0.008) or PMiT (Table S2a; *p* = 0.002) subtyping. They are also inversely related to the presence of nodular hyperplasia or additional neoplasms (other than mPTC) within the gland (Table S2a; *p* = 0.008 and *p* = 0.006, respectively), indicating that they represent the clinically relevant lesion in the thyroid. After multivariate analysis, each group also shows distinctive features in terms of both tumor growth and microscopic appearance (Figure 4, Table S2a,b, Figure 5, Figure 6 and Figure 7). The growth of group A tumors shows the following features: Infiltrative border (compared to group B, Table S2b; *p* = 0.001), intraglandular tumor spread (Table S2a; *p* = 0.002), presence of psammoma bodies in the surrounding parenchyma (Table S2a; *p* = 0.012), and vascular invasion (Table S2a; *p* = 0.025). 

We cannot prove that intraglandular tumor spread (neoplastic cell aggregates separated from the principal tumor mass by at least one layer of non-neoplastic thyroid) is the result of tumor cell growth inside blood or lymphatic vessels, since endothelial marker immunohistochemistry (CD31 and CD34) does not show reactivity around the neoplastic cell aggregates [13].

Group A mPTCs are often unicentric (compared to group B tumors that are more commonly multicentric, Table S2b; *p* = 0.003). The microscopic appearance of group A tumors is characterized by the presence of tall cell features (Table S2a; *p* = 0.006), an association with tumor fibrosis (Table S2a; *p* = <0.0001), the presence of psammoma bodies within the tumor (Table S2a; *p* = 0.005), and lack of a follicular growth pattern (Table S2a; *p* = 0.006).

Group B tumors show independent association with the following clinicopathologic parameters: Classical papillary carcinoma histotype (Table S2a; *p* = 0.02) and lack of additional neoplasms (other than mPTC) within the gland (Table S2a; *p* = 0.012). When compared to group A, group B tumors display borders that are less infiltrative (Table S2b; *p* = 0.001) and tend to be multicentric (Table S2b; *p* = 0.003), they have a lower proportion of tall cell features (Table S2b; *p* = 0.007), a lesser amount of tumor fibrosis (Table S2b; *p* = 0.02), and fewer psammoma bodies within the tumor (Table S2b; *p* = 0.04).

Group C tumors when compared to the most indolent mPTC group (group D) are more frequently associated with an intermediate as opposed to low ATA risk (Table S2a; *p* < 0.0001) and are inversely related to NIFTP (Table S2a; *p* = 0.045) or PMiT (Table S2a; *p* = 0.009) subtyping. Also, when compared to group D tumors, they are independently associated with infiltrative borders (Table S2a; *p* = 0.023) and tumor fibrosis (Table S2a, *p* = 0.016). When compared to the aggressive mPTC group (group A), they do not show intraglandular tumor spread (Table S2b; *p* = 0.001).

Group D mPTCs can often be subtyped as NIFTP (Table S2b; *p* = 0.008) or PMiT (Table S2b; *p* = 0.002), show an inverse relationship with intraglandular tumor spread (Table S2b; *p* = 0.002), psammoma bodies in the parenchyma surrounding the mPTC (Table S2b; *p* = 0.01), and with vascular invasion (Table S2b; *p* = 0.025). Microscopic features independently associated with group D are the follicular growth pattern (Table S2b; *p* = 0.006), scarcity of tall cells (Table S2b; *p* = 0.006), scarcity of tumor fibrosis (Table S2b; *p* < 0.0001), and scarcity of psammoma bodies with the tumor (Table S2b; *p* = 0.005).

## 3. Discussion

Papillary microcarcinomas are generally considered clinically indolent with relatively benign biologic features and an excellent prognosis [1,2]. Nevertheless, they represent a heterogeneous group, and include a subset capable of relatively aggressive behavior with a propensity to lymph node metastases, dissemination, and recurrence [13,14,15]. Current guidelines advise a reduction of diagnostic and therapeutic burden for low-risk thyroid cancers [16], to the point of avoiding surgical intervention in selected cases. Thus, accurate risk stratification of patients with mPTC has become essential for three important reasons: To avoid overtreatment, to select patients for active surveillance programs, and to enable the identification of the minority of tumors with the competence for aggressive behavior that need to be adequately managed to cure the patient [17]. 

We studied the precise histological location of the tumor with respect to the thyroid surface—a parameter never analyzed before—to discover whether the actual distance from the thyroid capsular surface correlates with specific clinicopathologic parameters, and whether it can be used as an additional tool for risk stratification.

Although papillary carcinomas can occur anywhere within the thyroid gland, we prove for the first time that the vast majority of mPTC arise peripherally, as suggested in the early articles from the 1950s [9]: The median distance of the tumor center from the thyroid surface is 3.5 mm.

The distribution of mPTC with respect to tumor size is random and that with respect to the distance of the edge of the tumor from the thyroid surface is skewed (most mPTCs reach close to the thyroid surface). However, when considering the distance of the tumor center (the origin of the tumor) from the surface of the thyroid, we find a normal, bell-shaped distribution, typical of naturally expected phenomena, suggesting that the peripheral section of the gland is predisposed to the development of mPTC. Ultrasound imaging does not always accurately classify nodules smaller than 3 to 5 mm [18], but we are aware of one sonographic study [19] that has provided measurements of the distance of the tumor center from the thyroid surface. The results are very similar to ours: Approximately 95% of nodules suspicious for mPTC were located at an average distance of 3 mm from the thyroid capsular surface [19].

We find that the site of origin of the tumor with respect to the thyroid surface correlates with important clinicopathologic parameters. We have thus identified four mPTC clusters. Large subcapsular mPTCs (group A: tumors size ≥5 mm and distance from the thyroid capsule = 0 mm) represent the group with the most worrisome characteristics, including the tall cell histotype, *BRAF* V600E mutation, tumor fibrosis, and aggressive growth with distinctively invasive features, such as infiltrative tumor border, intraglandular tumor spread, presence of psammoma bodies into the surrounding parenchyma, vascular invasion, and lymph node metastases. They are also the group with the largest proportion of persistent or recurrent disease and are strongly and independently associated with an intermediate (as opposed to low) ATA risk group by multivariate analysis.

According to uni- and multivariate analysis, the B, C, and D groups have a decreasing risk. Large and peripheral—but not subcapsular—tumors (Group B) are typically of the classic histotype, less invasive, with fewer lymph node metastases and with a lower proportion of tall cells and tumor fibrosis than group A tumors. Small subcapsular tumors (Group C), even if morphologically associated with infiltrative tumor borders and tumor fibrosis, do not feature intraglandular tumor spread, vascular invasion, or psammoma bodies in the surrounding parenchyma. At the bottom end of the spectrum, group D tumors (small, nonsubcapsular) represent the most favorable group. They are characterized by a follicular growth pattern, paucity of tall cells, lack of tumor fibrosis, and can frequently be subtyped as NIFTP [4,5] or PMiT [3], categories proposed for the most indolent mPTCs to avoid cancer overdiagnosis.

Several studies have identified prognostic factors predictive of aggressive mPTC behavior, but their prognostic relevance is still controversial. Features correlated with unfavorable outcomes are: Extensive fibrosis [20,21], age (<45 years), tumor multifocality, and lymph node metastasis at presentation [22]. *BRAF* V600E is the defining molecular feature of PTCs, which harbor the mutation in 40% to 80% of cases according to different series. Some studies have associated the mutation with prognostic factors generally related to aggressive behavior and with persistent disease in patients with mPTC [23,24,25,26,27]. Our group has carefully analyzed the microscopic, pathologic, and clinical aspects of *BRAF* V600E and *TERT*-mutated mPTCs, confirming that they are heterogeneous. Although *BRAF* V600E negative cases were clearly indolent tumors, the presence of the mutation was not a predictor of aggressive behavior in mPTC [12], while *TERT* promoter mutations did not correlate with unfavorable clinical features [28]. The location of the tumor immediately adjacent to the perithyroid adipose tissue has been recognized as an adverse prognostic factor [29]. This is not surprising, since peripheral tumors have by definition easy access to the extrathyroid soft tissues, including vascular and lymphatic spaces, as well as to critical perithyroid structures, such as the recurrent laryngeal nerve [29]. However, the impact of extrathyroid tumor extension (ETE) remains debatable: While some studies have associated microscopic ETE with tumor size and unfavorable prognosis [30,31,32], there is no definite correlation with reduced disease-specific survival [33] and microscopic ETE has been removed from the T3 stage of the AJCC 8th edition. [34,35]. 

Niemeier et al. have proposed a molecular-pathologic score based on a set of four parameters: Presence of *BRAF* V600E, tumor location (subcapsular, with no benign thyroid tissue between the tumor and the extrathyroid soft tissues), significant fibrosis, and intraglandular tumor spread to predict aggressive behavior (defined as lymph node metastases/tumor recurrence) [29], but with the exception of *BRAF* V600E that can be identified on preoperative cytology specimens, correct evaluation of the other parameters can only be performed on resected tumors. Nevertheless, our results strongly support the validity of the scoring system proposed by Niemeier [29]: Our group A tumors are subcapsular (by definition) and strongly associated by multivariate analysis with *BRAF* V600E, significant fibrosis, intraglandular tumor spread, and lymph node metastases. Furthermore, our data are consistent with clinical ultrasound studies that have shown how subcapsular tumors greater than 5 mm are more likely to have lymph node metastases [17,36,37].

In summary, this is the first study to accurately measure the origin of mPTCs with respect to the thyroid capsular surface and to propose an easy four-group risk subdivision of mPTC based on tumor size and location that can be applied before surgery. This could have an impact on clinical management, in refining the selection of thyroid nodules for preoperative fine needle aspiration, and in the proper selection of patients for active surveillance. Currently, while the ATA guidelines recommend performing FNA only for high-risk ultrasound nodules greater or equal to 1 cm, other guidelines apply less stringent criteria and suggest considering fine needle aspiration for those nodules in a subcapsular or paratracheal location [38]. At present, mPTCs that have a subcapsular location are considered appropriate (although not ideal) candidates for active surveillance programs [17]. Based on our findings, a careful approach is warranted for nodules ≥5 mm, attached to the thyroid capsule, and with irregular margins (group A tumors of this study). Independent of their potential extrathyroidal extension, these mPTCs are most likely associated with *BRAF* V600E, lymph node metastases, intermediate ATA recurrence risk, aggressive histotype (tall cell), and invasive features. Conversely, most other mPTCs, and particularly those that are small and nonsubcapsular (group D tumor of this study) may safely avoid aggressive preoperative screening and immediate surgical treatment.

## 4. Materials and Methods

### 4.1. Case Selection

The cases analyzed are part of an mPTC dataset previously reported [12]. As part of the project [12], histology slides and all pathologic and clinical data from six medical institutions covering different geographic areas of Italy (Bellaria and Maggiore Hospitals in Bologna, Northern Italy; University medical center-Sapienza Università di Roma in Rome, Central Italy; Casa Sollievo della Sofferenza Hospital in San Giovanni Rotondo, Southern Italy, the city Hospitals of Matera and Catanzaro, Southern Italy) were subjected to central review. This included microscopic review of hematoxylin and eosin (H&E)-stained histology sections by two pathologists with a special interest in thyroid pathology (GT and ADL) to define tumor diagnosis, tumor subtype, tumor stage, and all the variables analyzed in the study. All pathology reports and pertinent data from the patient charts were also reviewed to define the clinical and pathologic variables analyzed in the study. Inclusion criteria included: A diagnosis of microcarcinoma, availability of relevant clinicopathologic data, availability of H&E-stained histology slides for review of the case, and adequate material for *BRAF* V600E mutation analysis (see also Supplementary Materials). Human material was handled using anonymous codes and all samples were managed in compliance with the Helsinki Declaration. The study was approved by the ethics committee of the Sapienza Università di Roma on behalf of all the centers.

### 4.2. Location of the Microcarcinoma within the Thyroid

The measurement of the distance of the center of the tumor from the surface of the thyroid gland, the so-called thyroid capsule, was used to assess its subcapsular vs. nonsubcapsular location. The thyroid “capsule” is a thin fibrous layer that separates the gland from its surrounding stroma [6]. Although this layer can be poorly defined, particularly in the isthmus, the border of the thyroid parenchyma with the surrounding tissues is always evident on histology sections. The distance of the center of the tumor from the thyroid surface was calculated by adding the numerical values measured micrometrically of the edge of the tumor from the thyroid surface and of the radius of the tumor (Figure 1) (see also the Supplementary Materials).

### 4.3. Clinical and Pathologic Data

#### 4.3.1. Clinical Data

Stage was defined according to current criteria (American Joint Committee on Cancer, 2017) [34]. Patients were retrospectively assigned to low or intermediate recurrence risk groups following American Thyroid Association criteria (2015) [39]. Unfavorable tumor-related events were classified as persistent disease (documented within one year of the diagnosis) and recurrent disease (documented later than one year after the diagnosis). Disease status was classified as follows: “Excellent response” when there was no biochemical (abnormal thyroglobulin values), structural (imaging studies) or clinical evidence of disease; and “structural incomplete response”, when imaging studies indicated evidence of disease. “Biochemical incomplete response” or “indeterminate response” were not considered as evidence of disease [33] (see also the Supplementary Materials).

#### 4.3.2. Tumor Subtype

According to the World Health Organization (2017) criteria, we considered mPTC a papillary carcinoma with a size smaller or equal to one centimeter, regardless of whether the tumor was discovered incidentally or following a targeted fine needle aspiration procedure [40]. Tumor subtypes were defined according to criteria used for tumors larger than one centimeter [40] (see also the Supplementary Materials). 

#### 4.3.3. Characteristics of Tumor Growth

See the Supplementary Materials for the definition of the variables analyzed in Table 2, Table S1, and Table S2a,b. Intraglandular tumor spread was defined as the presence of neoplastic cell aggregates separated from a principal tumor mass by at least one layer of non-neoplastic thyroid. For the purpose of this study, tumor multicentricity was defined as the presence of at least two separate tumor foci of similar dimensions, irrespective of the histologic appearance of the tumor, or the presence of at least two separate tumor foci of different morphology, regardless of their diameter. Since, according to the above definition, multicentric thyroid microcarcinomas may represent synchronous independent tumors, multicentric microcarcinoma foci were analyzed separately.

#### 4.3.4. Microscopic Appearance of the Tumor

See the Supplementary Materials for the definition of the variables analyzed in Table 2, Table S1, and Table S2a,b.

### 4.4. Immunohistochemistry

Immunohistochemistry for Cytokeratin 19, CD31, CD34, Podoplanin, and *BRAF* V600E-specific antibody (clone VE1) was used to highlight infiltrative tumor borders, intraglandular tumor spread, and vascular invasion. It was performed in selected cases using a Ventana Benchmark platform according to previously published protocols [41] (see also the Supplementary Materials).

### 4.5. BRAF V600E Mutation Analysis

Mutation analysis was performed using an Allele Specific Locked Nucleic Acid PCR method, according to previously published protocols from formalin-fixed paraffin-embedded material microdissected from tumor tissue [42] (see also the Supplementary Materials).

### 4.6. Statistical Analysis

Summary statistics were reported as numbers (percentages) or mean ± standard deviation. Categorical variables were analyzed with the X^2^ test or the Fisher exact test, as appropriate. Continuous variables were compared between groups using the Student t test or one-way analysis of variance and Bonferroni multiple-comparison test. Univariate and multivariate conditional logistic regression models were performed in order to analyze the relationship between variables and mPTC groups. Conditional logistic regression models were conducted on pertinent clinicopathologic variables that reached *p* < 0.2 at univariate analysis. Model building followed a backward-stepwise approach, and the test of term significance was the Wald chi-square test. 

Stata SE 14.2 for Windows (StataCorpLp, College Station, TX, USA) and GraphPad Prism, (GraphPad Software, San Diego, CA, USA) software were used for statistical analyses. All *p* values refer to two-tailed tests of significance; *p* < 0.05 was considered statistically significant.

## 5. Conclusions

This study accurately measured the origin of mPTCs with respect to the thyroid capsular surface for the first time: mPTCs arise at a median distance of 3.5 mm (range 0.5–11 mm) below the surface of the thyroid gland. We found that the clinicopathologic features of mPTC vary according to the tumor size and distance from the thyroid surface and proposed an easy four-group risk subdivision of mPTC based on the tumor size and location that can be applied before surgery. Based on our findings, a careful approach is warranted for nodules ≥5 mm, attached to the thyroid capsule, and with irregular margins (group A tumors of our study). This four-mPTC group model may have an impact on clinical management, in refining the selection of thyroid nodules for preoperative fine needle aspiration, and in the proper selection of patients for active surveillance. 

## Figures and Tables

**Table 1:** Clinicopathologic characteristics (Total: 286 mPTCs).

Age (years)	50.2 ± 13 ^a^
Female sex	218 (76.2%)
Tumor diagnosis	
Follicular variant	124 (43.4%)
Classic variant	110 (38.5%)
Tall cell variant	36 (12.6%)
Other histotypes	16 (5.6%)
Tumor size (mm)	5.2 ± 2.8 ^a^
Distance of the tumor center from the thyroid capsule	3.5 (0.5–8.5) ^b^
*BRAF* V600E mutation	140 (48.9%)
Lymph node metastasis	29 (10.1%)
ATA (2015) recurrence risk groups	
Low risk	200 (69.9%)
Intermediate risk	86 (30.1%)
AJCC stage (8th ed) ^c^	
I	268 (93.7%)
II	8 (6.3%)
Unfavorable disease-related patient events (persistent or recurrent disease)	8 (2.8%)
Incidental findings	173 (58%)
Aggressive growth (IGTS-infiltrative border-extrathyroidal extension)	195 (65.4%)

^a^ mean + standard deviation; ^b^ median and range; ^c^ in 10 cases the stage could not be determined.

**Table 2:** Univariate analysis comparing the four microcarcinoma groups (Total: 286 mPTC).

Variables	GROUP A	GROUP B	GROUP C	GROUP D	*p*-Value
	Subcapsular & ≥ 5 mm n = 93 (32.5%) n (%)	Nonsubcapsular & ≥ 5 mm n = 54 (18.9%) n (%)	Subcapsular & < 5 mm n = 41 (14.3%) n (%)	Nonsubcapsular & < 5 mm n = 98 (34.3) n (%)	
**Clinicopathologic Features**					
Age (mean ± s.d.)	47.3 ± 12.8	52.6 ± 15.1	51 ± 12.2	51.7 ± 12.1	0.057 ^a^
Female sex (n = 218)	72 (77.4%)	38 (70.4%)	31 (75.6%)	77 (78.6%)	0.811 ^a^
*Pathological diagnosis*					<0.0001 ^a^
Follicular variant (n = 124)	18 (19.4%)	21 (38.9%)	23 (56.1%)	62 (63.3%)	
Classic variant (n = 110)	45 (48.4%)	27 (50.0%)	11 (26.8%)	27 (27.6%)	
Tall cell variant (n = 36)	24 (25.8%)	2 (3.7%)	5 (12.2%)	5 (5.1%)	
Other histotypes (n = 16)	6 (6.5%)	4 (7.4%)	2 (4.9%)	4 (4.1%)	
NIFTP (n = 50)	4 (4.3%)	11 (20.4%)	5 (12.2%)	30 (30.6%)	<0.0001 ^a^
PMiT (n = 69)	10 (10.8%)	14 (25.9%)	5 (12.2%)	40 (40.8%)	<0.0001 ^a^
*BRAF* V600E mutation (n = 140)	64 (68.8%)	25 (46.3%)	20 (48.8%)	36 (36.7%)	<0.0001 ^a^
Lymph node metastasis (n = 29)	18 (19.4%)	5 (9.3%)	1 (2.4%)	5 (5.1%)	0.003 ^a^
AJCC stage (8th ed.) ^c^					0.734 ^a^
I (n = 268)	84 (90.3%)	51 (94.4%)	38 (92.7%)	95 (96.9%)	
II (n = 8)	4 (4.3%)	1 (1.9%)	1 (2.4%)	2 (2.0%)	
ATA (2015) recurrence risk groups					<0.0001 ^a^
Low risk (n = 200)	41 (44.1%)	46 (85.2%)	23 (56.1%)	90 (91.8%)	
Intermediate risk (n = 86)	52 (55.9%)	8 (14.8%)	18 (43.9%)	8 (8.2%)	
Other thyroid neoplasms (n = 32)	5 (5.4%)	1 (1.9%)	7 (17.1%)	19 (19.4%)	0.001 ^a^
Hyperthyroidism (n = 10)	1 (1.1%)	2 (3.7%)	3 (7.3%)	4 (4.1%)	0.321 ^a^
Nodular hyperplasia (n = 181)	43 (46.2%)	38 (70.4%)	29 (70.7%)	71 (72.4%)	0.001 ^a^
Lymphocytic thyroiditis (n = 35)	10 (10.8%)	4 (7.4%)	6 (14.6%)	15 (15.3%)	0.486 ^a^
Incidental findings (n = 169)	55 (59.1%)	37 (68.5%)	32 (78.0%)	78 (79.6%)	0.006 ^a^
Administration of RAI (n = 114)	48 (51.6%)	27 (50.0%)	12 (29.3%)	27 (27.6%)	0.005 ^a^
Unfavorable disease-related patient events (persistent or recurrent disease) (n = 8)	5 (5.4%)	2 (3.7%)	0 (0%)	1 (1.0%)	0.265 ^a^
**Characteristics of Tumor Growth**					
Cystic component (n = 18)	7 (7.5%)	8 (14.8%)	1 (2.4%)	2 (2.0%)	0.012 ^a^
Invasion of extrathyroidal tissues (n = 68)	50 (53.8%)	0 (0%)	18 (43.9%)	0 (0%)	<0.0001 ^a^
Infiltrative tumor border (n = 171)	72 (77.4%)	21 (38.9%)	29 (70.7%)	49 (50.0%)	<0.0001 ^a^
High-grade features (mitoses and/or necrosis) (n = 54)	25 (26.9%)	12 (22.2%)	7 (17.1%)	10 (10.2%)	0.029 ^a^
Mitoses (n = 52)	24 (25.8%)	11 (20.4%)	7 (17.1%)	10 (10.2%)	0.049 ^a^
Tumor necrosis (n = 7)	5 (5.4%)	1 (1.9%)	1 (2.4%)	0 (0%)	0.12 ^a^
Vascular invasion (n = 17)	15 (16.1%)	1 (1.9%)	0 (0%)	1 (1.0%)	<0.0001 ^a^
Microcarcinoma multicentric (n = 106)	25 (26.9%)	27 (50.0%)	18 (43.9%)	36 (36.7%)	0.032^a^
Intraglandular tumor spread (n = 112)	56 (60.2%)	20 (37.0%)	10 (24.4%)	25 (25.5%)	<0.0001 ^a^
Psammoma bodies out of the carcinoma (n = 31)	19 (20.4%)	6 (11.1%)	2 (4.9%)	4 (4.1%)	0.002 ^a^
Intratumoral lymphoid cells (n = 59)	29 (31.2%)	14 (25.9%)	5 (12.2%)	11 (11.2%)	0.003 ^a^
Peritumoral lymphoid cells (n = 53)	29 (31.2%)	10 (18.5%)	7 (17.1%)	7 (7.1%)	<0.0001 ^a^
**Microscopic Appearance of Papillary Microcarcinoma**					
*Nuclei*					
Pseudoinclusions (n = 45)	25 (26–9%)	8 (14.8%)	3 (7.3%)	9 (9.2%)	0.003 ^a^
Grooves (n = 167)	64 (68.8%)	35 (64.8%)	25 (61.0%)	43 (43.9%)	0.004 ^a^
Nuclear membrane irregularities (n = 207)	82 (88.2%)	36 (66.7%)	28 (68.3%)	61 (62.2%)	0.0001 ^a^
Optically clear nuclei (n = 207)	68 (73.1%)	38 (70.4%)	26 (63.4%)	75 (76.5%)	0.207 ^a^
*Cytoplasm*					
Cells with cytoplasmic eosinophilia (n = 159)	57 (61.3%)	32 (59.3%)	26 (63.4%)	44 (44.9%)	0.012 ^a^
Cells with cytoplasmic clearing (n = 63)	27 (29.0%)	11 (20.3%)	9 (22.0%)	16 (16.3%)	0.217 ^a^
Tall cells (n = 62)	36 (38.7%)	10 (18.5%)	9 (22.0%)	7 (7.1%)	<0.0001^a^
Tall cell features (mean% ± s.d.)	25.6 ± 36.5	8.3 ± 21	14.4 ± 31	5.1 ± 19.7	0.0001 ^b^
Psammoma bodies within the carcinoma (n = 31)	40 (43.0%)	11 (20.4%)	5 (12.2%)	11 (11.2%)	<0.0001 ^a^
Fibrosis associated with the tumor (n = 83)	42 (45.2%)	12 (22.2%)	14 (34.1%)	15 (15.3%)	<0.0001 ^a^
*Tumor patterns (mean% ± s.d.)*					
Papillary	40.4 ± 33.5	33.8 ± 39.6	22.9 ± 35	19.7 ± 31.5	0.0003 ^b^
Follicular	44.4 ± 32.8	54.8 ± 38.3	64 ± 39.4	73.4 ± 35.7	<0.0001 ^b^
Solid/trabecular	15.2 ± 19.7	11.4 ± 21.1	13 ± 25.9	6.9 ± 16.9	0.039 ^b^

a: Pearson’s chi-squared test; b: Kruskal–Wallis test; c: In 10 cases, the stage could not be determined.

## Supplementary Materials

The following are available online at https://www.mdpi.com/2072-6694/12/1/246/s1, Table S1: Univariate analysis—Distance of the tumor center, Table S2: Multinomial logistic regression analysis comparing the four microcarcinoma groups. Figure S1: Distribution of microcarcinomas according to size and to the distance of their edge from the thyroid capsule, Figure S2: Distribution of microcarcinoma groups according the distance of the tumor center from the thyroid surface. Supplemental Materials and Methods.
