# Supplementary material for: Does the Site of Origin of the Microcarcinoma with Respect to the Thyroid Surface Matter? A Multicenter Pathologic and Clinical Study for Risk Stratification
Source: Cancers (Basel). 2020 Jan 19;12(1):246. doi: 10.3390/cancers12010246 (PMC7016743; doi:10.3390/cancers12010246)
Supplement: Supplementary file 1 [file cancers-12-00246-s001.zip › 2.cancers-683690-Supplementary Figures and Table1.docx]

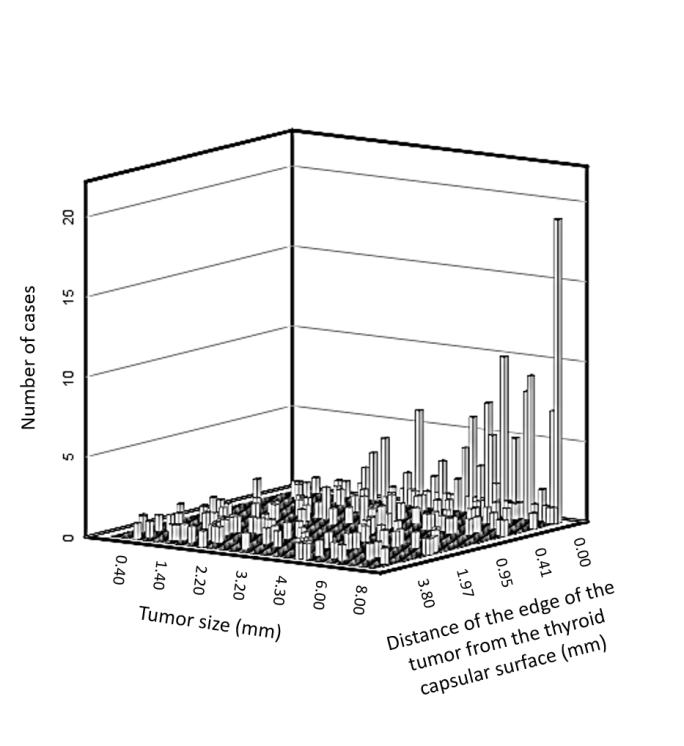


**Figure S1.** Distribution of microcarcinomas according to size and to the distance of their edge from the thyroid capsule.


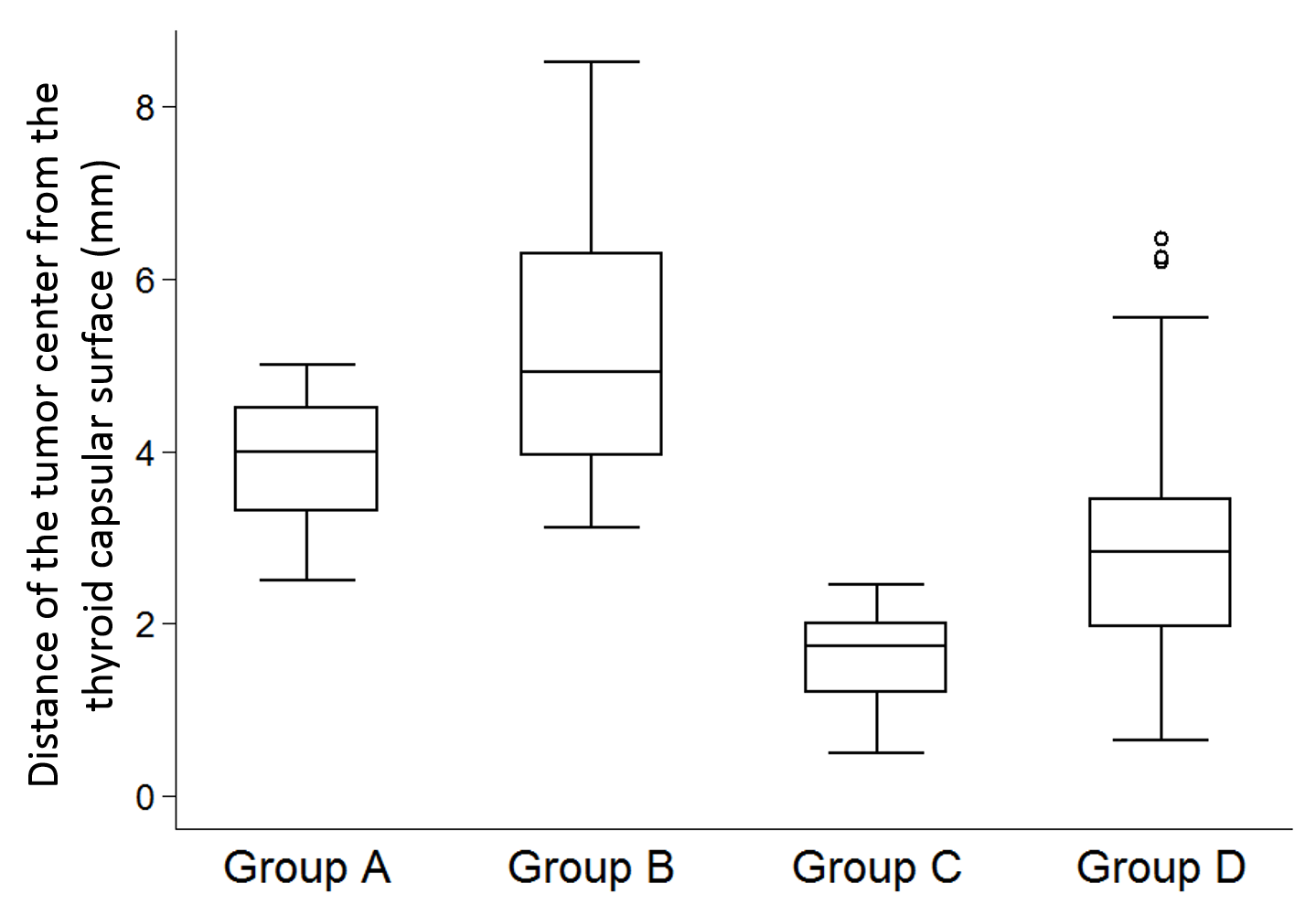


**Figure S2.** Distribution of microcarcinoma groups according the distance of the tumor center from the thyroid surface. Group A, large subcapsular mPTC: size > or = 5 mm and distance of the edge of the tumor from the thyroid capsule = 0 mm; Group B, large nonsubcapsular mPTC: size > or = 5 mm and distance of the edge of the tumor from the thyroid capsule > 0 mm; Group C, small subcapsular mPTC: size < 5 mm and distance of the edge of the tumor from the thyroid capsule = 0 mm; Group D, small nonsubcapsular mPTC: size < 5 mm and distance of the edge of the tumor from the thyroid capsule > 0 mm.

**Table S1.** Analysis.

| Variables | | Distance of the tumor center | | Univariate analysis | |
| --- | --- | --- | --- | --- | --- |
|  |  | median (min, max) | | P-value | |
| All cases (n=286) | | 3.5 (0.5-11.0) | |  | |
| **Clinicopathologic features** | | | | | |
| Age | | | | | |
| <55 years (n= 172) | | 3.36 (0.5-7.75) | | 0.942 | |
| ≥ 55 years (n= 104) | | 3.5 (0.65-8.5) | |  |  |
| Sex (male vs female) | | | | | |
| Male (n= 61) | | 3.85 (0.75-7.55) | | 0.085 | |
| Female (n= 218) | | 3.325 (0.5-8.5) | |  |  |
| Pathological diagnosis | | | | | |
| Follicular variant (n= 124) | | 2.89 (0.5-8) | | 0.0003 | |
| Classic variant (n=110) | | 3.76 (0.85-8.5) | |  |  |
| Tall cell variant (n= 36) | | 3.9 (1.1-6.25) | |  |  |
| Other histotypes (n= 16) | | 3.67 (1.47-6.4) | |  |  |
| NIFTP | | | | | |
| Absent (n= 234) | | 3.5 (0.5-8.5) | | 0.125 | |
| Present (n= 51) | | 2.95 (0.7-7.25) | |  |  |
| PMiT | | | | | |
| Absent (n= 217) | | 3.52 (0.5-8.5) | | 0.05 | |
| Present (n= 69) | | 2.95 (0.65-8) | |  |  |
| *BRAF* V600E mutation | | | | | |
| WT (n= 146) | | 3.15 (0.5-8.5 | | 0.027 | |
| Mutated (n= 140) | | 3.75 (0.85-7.75) | |  |  |
| Lymph node metastasis | | | | | |
| Negative (n= 257) | | 3.35 (0.5-8.25) | | 0.79 | |
| Positive (n= 29) | | 4 (1.25-8.5) | |  |  |
| AJCC stage (8th ed.) | | | | | |
| I (n= 268) | | 3.45 (0.5-8.25) | | 0.71 | |
| II (n= 8) | | 3.65 (1.25-8.5) | |  |  |
| ATA (2015) recurrence risk groups | | | | | |
| Low risk (n= 200) | | 3.35 (0.5-8.25) | | 0.61 | |
| Intermediate risk (n= 86) | | 3.5 (1-8.5) | |  |  |
| Other thyroid neoplasms | | | | | |
| Absent (n= 254) | | 3.5 (0.5-8.5) | | 0.0037 | |
| Present (n= 32) | | 2.6 (0.65-7.5) | |  |  |
| Hyperthyroidism | |  | |  | |
| Absent (n= 276) | | 3.5 (0.5-8.5) | | 0.188 | |
| Present (n= 10) | | 2.65 (0.95-5.4) | |  |  |
| Nodular hyperplasia | |  | |  | |
| Absent (n= 105) | | 3.5 (0.65-8.5) | | 0.087 | |
| Present (n= 181) | | 3.22 (0.5-8.25) | |  |  |
| Lymphocytic thyroiditis | |  | |  | |
| Absent (n= 251) | | 3.5 (0.65-8.5) | | 0.104 | |
| Present (n= 35) | | 2.95 (0.5-8.25) | |  |  |
| Incidental findings | | | | | |
| No (n= 103) | | 3.5 (0.5-8.25) | | 0.353 | |
| Yes (n= 169) | | 3.45 (0.65-8.5) | |  |  |
| Administration of RAI | | | | | |
| Absent (n= 97) | | 3.25 (0.5-7.75) | | 0.067 | |
| Present (n= 114) | | 3.75 (0.7-8.5) | |  |  |
| Unfavorable disease-related patient events (persistent or recurrent disease) | | | | | |
| No events (n= 199) | | 3.5 (0.5-8.5) | | 0.0152 | |
| Events (n= 8) | | 4.67 (3.65-5.4) | |  |  |
| **Characteristics of tumor growth** | | | | | |
| Cystic component | | | | | |
| Absent (n= 268) | | 3.41 ( 0.5-8) | | 0.0093 | |
| Present (n= 18) | | 4.37 (1.9-8.5) | |  |  |
| Infiltrative tumor border | | | | | |
| Absent (n= 115) | | 3.25 (0.7-8.5) | | 0.826 | |
| Present (n= 171) | | 3.5 (0.5-8.25) | |  |  |
| High-grade features (mitoses and/or necrosis) | | | | | |
| Absent (n= 231) | | 3.3 (0.5-8.5) | | 0.0022 | |
| Present (n= 54) | | 4.05 (1.5-7.5) | |  |  |
| Mitoses | | | | | |
| Absent (n= 233) | | 3.3 (0.5-8.5) | | 0.0056 | |
| Present (n= 52) | | 4.05 (1.5-6.45) | |  |  |
| Necrosis | | | | | |
| Absent (n= 278) | | 3.45 (0.5-8.5) | | 0.186 | |
| Present (n= 7) | | 4.35 (2-7.5) | |  |  |
| Vascular invasion | | | | | |
| Absent (n= 269) | | 3.35 (0.5-8.5) | | 0.004 | |
| Present (n= 17) | | 4.5 (2.5-7.5) | |  |  |
| Microcarcinoma multicentric | | | | | |
| Absent (n= 178) | | 3.45 (0.65-8.5) | | 0.875 | |
| Present (n= 106) | | 3.5 (0.5-8.25) | |  |  |
| Intraglandular tumor spread | | | | | |
| no (n= 174) | | 3 (0.5-8.25) | | <0.0001 | |
| yes (n= 112) | | 3.86 (1.2-8.5) | |  |  |
| Psammoma bodies out of the carcinoma | |  | |  | |
| Absent (n= 255) | | 3.3 (0.5-8.5) | | 0.004 | |
| Present (n= 31) | | 4 (1.85-7.25) | |  |  |
| Intratumoral lymphoid cells | | | | | |
| Absent (n= 226) | | 3.25 (0.5-8) | | 0.0054 | |
| Present, few (n= 42) | | 4 (1-8.5) | |  |  |
| Present, diffuse (n= 17) | | 4.25 (1.7-7.75) | |  |  |
| Peritumoral lymphoid cells | | | | | |
| Absent (n= 150) | | 3.1 (0.5-8) | | 0.0314 | |
| Present, few (n= 82) | | 3.6 (0.7-7.75) | |  |  |
| Present, diffuse (n= 53) | | 3.95 (1.1-8.5) | |  |  |
| **Microscopic appearance of papillary microcarcinoma** | | | | | |
| Nuclei | | | | | |
| Pseudoinclusions | | | | | |
| Absent (n= 240) | | 3.3 (0.5-8.5) | | 0.0271 | |
| Present (n= 45) | | 4 (1.1-7.75) | |  |  |
| Grooves (0,1,2) | | | | | |
| Absent (n= 118) | | 3.24 (0.65-8) | | 0.023 | |
| Present, few (n= 135) | | 3.5 (0.5-8.5) | |  |  |
| Present, diffuse (n= 32) | | 4 (1.75-7.75) | |  |  |
| Nuclear membrane irregularities | | | | | |
| Absent (n= 78) | | 2.9 (0.5-8) | | 0.0058 | |
| Present, few (n= 123) | | 3.5 (0.65-7.25) | |  |  |
| Present, diffuse (n= 84) | | 3.75 (0.95-8.5) | |  |  |
| Optically clear nuclei | | | | | |
| Absent (n= 78) | | 3.75 (0.5-7-75) | | 0.27 | |
| Present, few (n= 129) | | 3.25 (0.65-8.5) | |  |  |
| Present, diffuse (n= 78) | | 3.5 (0.75-8.25) | |  |  |
| Cytoplasm | | | | | |
| Cells with cytoplasmic eosinophilia | | | | | |
| Absent (n= 126) | | 3.25 (0.65-8) | | 0.003 | |
| Present, few (n= 63) | | 3.375 (0.5-7-55) | |  |  |
| Present, diffuse (n= 96) | | 3.96 (1.1-8.5) | |  |  |
| Cells with cytoplasmic clearing | | | | | |
| Absent (n= 222) | | 3.5 (0.5-8.5) | | 0.039 | |
| Present, few (n= 51) | | 3.22 (1.2-6.5) | |  |  |
| Present, diffuse (n= 12) | | 5 (1.85-7.75) | |  |  |
| Tall cells (absent, present) | | | | | |
| Absent (n= 223) | | 3.25 (0.5-8.5) | | 0.0025 | |
| Present (n= 62) | | 4 (1.1-7.75) | |  |  |
| Psammoma bodies within the carcinoma | | | | | |
| Absent (n= 255) | | 3.3 (0.5-8.5) | | 0.0039 | |
| Present (n= 31) | | 4 (1.85-7.25) | |  |  |
| Fibrosis associated with the tumor | | | | | |
| Absent (n= 202) | | 3.25 (0.65-8.25) | | 0.0623 | |
| Present (n= 83) | | 3.75 (0.5-8.5) | |  |  |
| Tumor growth patterns | |  | |  | |
| Papillary | | | | | |
| < 50% (n= 198) | | 3.3 (0.5-8.25) | | 0.004 | |
| ≥ 50% (n= 87) | | 3.85 (1.1-8.5) | |  |  |
| Follicular | | | | | |
| < 50% (n= 113) | | 3.75 (1.1-8.5) | | 0.008 | |
| ≥ 50% (n= 172) | | 3.24 (0.5-8.25) | |  |  |
| Solid/trabecular | | | | | |
| < 50% (n= 240) | | 3.35 (0.5-8.5) | | 0.155 | |
| ≥ 50% (n= 45) | | 3.75 (1.1-8) | |  |  |
