# Supplementary material for: Does the Site of Origin of the Microcarcinoma with Respect to the Thyroid Surface Matter? A Multicenter Pathologic and Clinical Study for Risk Stratification
Source: Cancers (Basel). 2020 Jan 19;12(1):246. doi: 10.3390/cancers12010246 (PMC7016743; doi:10.3390/cancers12010246)
Supplement: Supplementary file 1 [file cancers-12-00246-s001.zip › 3.cancers-683690-Supplementary Table S2.docx]

| **Table S2a - Multivariate Four Groups** | | | | | | | | | |
| --- | --- | --- | --- | --- | --- | --- | --- | --- | --- |
|  |  |  |  |  |  |  |  |  |  |
| **Multinomial logistic regression analysis comparing the four microcarcinoma groups (Total: 286 mPTCs; indolent Group D tumors as reference)** | | | | | | | | | |
| Variables | **GROUP A** | | | **GROUP B** | | | **GROUP C** | | |
|  | **Subcapsular and ≥ 5 mm** | | | **Nonsubcapsular and ≥ 5 mm** | | | **Subcapsular and < 5 mm** | | |
|  | RRR | P-value | 95% CI | RRR | P-value | 95% CI | RRR (S.E.) | P-value | 95% CI |
| **Clinicopathologic features** | | | | | | | | | |
| Pathological diagnosis |  |  |  |  |  |  |  |  |  |
| Classic variant ^a^ | 3.32 | 0.001 | 1.60-6.91 | 2.41 | 0.02 | 1.13-5.14 | 1.02 | 0.95 | 0.42-2.49 |
| Tall cell variant ^a^ | 10.59 | <0.0001 | 3.45-32.53 | 0.94 | 0.94 | 0.17-5.25 | 2.62 | 0.16 | 0.69-9.92 |
| NIFTP ^b^ | 0.21 | 0.008 | 0.07-0.66 | 0.69 | 0.42 | 0.29-1.69 | 0.31 | 0.045 | 0.10-0.97) |
| PMiT ^c^ | 0.26 | 0.002 | 0.11-0.61 | 0.64 | 0.26 | 0.29-1.39 | 0.24 | 0.009 | 0.08-0.70 |
| *BRAF* V600E mutation ^d^ | 3.32 | <0.0001 | 1.72-6.43 | 1.27 | 0.52 | 0.61-2.64 | 2.05 | 0.07 | 0.94-4.49 |
| Lymph node metastasis ^d^ | 3.95 | 0.028 | 1.16-13.50 | 2.35 | 0.24 | 0.56-9.34 | 0.55 | 0.60 | 0.06-5.16 |
| Other thyroid neoplasms ^d^ | 0.21 | 0.006 | 0.07-0.64 | 0.07 | 0.012 | 0.01-0.57 | 0.75 | 0.59 | 0.27-2.11 |
| Nodular hyperplasia ^d^ | 0.40 | 0.008 | 0.20-0.78 | 0.85 | 0.69 | 0.39-1.87 | 1.09 | 0.85 | 0.46-2.59 |
| ATA (2015) recurrence risk groups ^e^ | 10.46 | <0.0001 | 4.19-26.12 | 1.72 | 0.34 | 0.57-5.18 | 8.42 | <0.0001 | 2.98-23.81 |
| **Characteristics of tumor growth** | | | | | | | | | |
| Infiltrative tumor border | 1.79 | 0.118 | 0.86-3.71 | 0.41 | 0.025 | 0.19-0.90 | 2.61 | 0.023 | 1.14-5.98 |
| Microcarcinoma multicentric | 0.53 | 0.068 | 0.26-1.04 | 1.65 | 0.15 | 0.83-3.31 | 1.19 | 0.66 | 0.56-2.53 |
| Intraglandular tumor spread | 3.12 | 0.002 | 1.54-6.32 | 2.19 | 0.062 | 0.96-4.99 | 0.63 | 0.31 | 0.25-1.55 |
| Psammoma bodies out of the carcinoma | 4.64 | 0.012 | 1.41-15.28 | 2.12 | 0.27 | 0.55-8.26 | 1.33 | 0.75 | 0.23-7.75 |
| Vascular invasion | 10.98 | 0.025 | 1.34-89.90 | 2.27 | 0.57 | 0.13-38.67 | 6.09 | 0.98 | 0-. |
| **Microscopic appearance of papillary microcarcinoma** | | | | | | | | | |
| Tall cell features | 1.02 | 0.006 | 1.01-1.03 | 1.00 | 0.98 | 0.98-1.02 | 1.01 | 0.13 | 1.00-1.03 |
| Fibrosis associated with the tumor | 3.88 | <0.0001 | 1.84-8.18 | 1.56 | 0.31 | 0.66-3.70 | 2.86 | 0.016 | 1.21-6.75 |
| Psammoma bodies within the carcinoma | 2.24 | 0.005 | 1.28-3.91 | 1.26 | 0.49 | 0.66-2.40 | 0.78 | 0.58 | 0.33-1.87 |
| Tumor pattern follicular | 0.99 | 0.006 | 0.98-1.00 | 0.99 | 0.01 | 0.98-1.00 | 0.99 | 0.43 | 0.98-1.00 |
|  |  |  |  |  |  |  |  |  |  |
| Multinomial logistic regression considering as reference Group D tumors (mPTC subcapsular and < 5 mm); RRR–relative risk ratio; S.E.-Standard Error; 95% CI–95% confidence interval.  Multivariate models excluding variables that might be considered co-dependent:  a: without ATA, stage and BRAF, but with pathological diagnosis, adjusted for pertinent variables (*BRAF* V600E, lymph node metastasis, other thyroid neoplasms, nodular hyperplasia)  b: without ATA, stage, pathological diagnosis but with NIFTP subtype, adjusted for pertinent variables (*BRAF* V600E, other thyroid neoplasms, nodular hyperplasia)  c: without ATA, stage, pathological diagnosis, but with PMiT subtype, adjusted for pertinent variables (*BRAF* V600E, lymph node metastasis, other thyroid neoplasms, nodular hyperplasia)  d: without ATA, stage and pathological diagnosis  e: with ATA, without stage and pathological diagnosis, adjusted for pertinent variables (*BRAF* V600E, other thyroid neoplasms, nodular hyperplasia) | | | | | | | | | |

| **Table S2b - Multivariate Four Groups** | | | | | | | | | | | | |
| --- | --- | --- | --- | --- | --- | --- | --- | --- | --- | --- | --- | --- |
|  |  |  |  |  |  |  |  |  |  |  |  |  |
| **Multinomial logistic regression analysis comparing the four microcarcinoma groups (Total: 286 mPTCs; aggressive Group A tumors as reference)** | | | | | | | | | | | | |
| Variables | **GROUP B** | | | **GROUP C** | | | | **GROUP D** | | | | |
|  | **Nonsubcapsular and ≥ 5 mm** | | | **Subcapsular and < 5 mm** | | | | **Nonsubcapsular and < 5 mm** | | | | |
|  | RRR | P-value | 95% CI | RRR | P-value | | 95% CI | RRR | P-value | | 95% CI | |
| **Clinicopathologic features** | | | | | | | | | | | | |
| Pathological diagnosis |  |  |  |  |  |  | |  |  | |  | |
| Classic variant ^a^ | 0.72 | 0.43 | 0.33-1.61 | 0.31 | 0.015 | 0.12-0.79 | | 0.30 | 0.001 | | 0.15-0.63 | |
| Tall cell variant ^a^ | 0.09 | 0.003 | 0.02-0.43 | 0.25 | 0.019 | 0.08-0.79 | | 0.09 | <0.0001 | | 0.03-0.29 | |
| NIFTP ^b^ | 3.29 | 0.06 | 0.92-11.76 | 1.45 | 0.62 | 0.33-6.40 | | 4.73 | 0.008 | | 1.51-14.84 | |
| PMiT ^c^ | 2.42 | 0.07 | 0.93-6.27 | 0.92 | 0.89 | 0.27-3.05 | | 3.80 | 0.002 | | 1.64-8.81 | |
| *BRAF* V600E mutation ^d^ | 0.38 | 0.010 | 0.18-0.80 | 0.62 | 0.25 | 0.27-1.40 | | 0.30 | <0.0001 | | 0.16-0.58 | |
| Lymph node metastasis ^d^ | 0.60 | 0.36 | 0.20-1.80 | 0.14 | 0.06 | 0.02-1.12 | | 0.25 | 0.028 | | 0.07-0.86 | |
| Other thyroid neoplasms ^d^ | 0.34 | 0.34 | 0.04-3.09 | 3.54 | 0.06 | 0.96-12.97 | | 4.71 | 0.006 | | 1.57-14.13 | |
| Nodular hyperplasia ^d^ | 2.14 | 0.048 | 1.01-4.55 | 2.74 | 0.02 | 1.15-6.48 | | 2.51 | 0.008 | | 1.28-4.93 | |
| ATA (2015) recurrence risk groups ^e^ | 0.16 | <0.0001 | 0.07-0.40 | 0.80 | 0.61 | 0.35-1.86 | | 0.10 | <0.0001 | | 0.04-0.24 | |
| **Characteristics of tumor growth** | | | | | | | | | | | | |
| Infiltrative tumor border | 0.23 | 0.001 | 0.10-0.53 | 1.46 | 0.42 | | 0.58-3.68 | 0.56 | | 0.12 | 0.27-1.16 | |
| Microcarcinoma multicentric | 3.15 | 0.003 | 1.46-6.79 | 2.25 | 0.056 | | 0.98-5.18 | 1.90 | | 0.068 | 0.95-3.78 | |
| Intraglandular tumor spread | 0.70 | 0.4 | 0.31-1.61 | 0.20 | 0.001 | | 0.08-0.50 | 0.32 | | 0.002 | 0.16-0.65 | |
| Psammoma bodies out of the carcinoma | 0.46 | 0.17 | 0.15-1.40 | 0.29 | 0.12 | | 0.06-1.37 | 0.21 | | 0.01 | 0.06-0.71 | |
| Vascular invasion | 0.21 | 0.14 | 0.02-1.71 | 5.54 | 0.98 | | 0-. | 0.09 | | 0.025 | 0.01-0.74 | |
| **Microscopic appearance of papillary microcarcinoma** | | | | | | | | | | | | |
| Tall cell features | 0.98 | 0.007 | 0.97-0.99 | 0.99 | 0.34 | | 0.98-1.00 | 0.98 | 0.006 | | | 0.97-0.99 |
| Fibrosis associated with the tumor | 0.40 | 0.02 | 0.18-0.89 | 0.74 | 0.46 | | 0.33-1.65 | 0.26 | <0.0001 | | | 0.12-0.54 |
| Psammoma bodies within the carcinoma | 0.56 | 0.04 | 0.32-0.98 | 0.35 | 0.01 | | 0.16-0.78 | 0.45 | 0.005 | | | 0.26-0.78 |
| Tumor pattern follicular | 1.00 | 0.91 | 0.99-1.01 | 1.01 | 0.15 | | 1.00-1.02 | 1.01 | 0.006 | | | 1.00-1.02 |
|  |  |  |  |  |  | |  |  |  | | |  |
| Multinomial logistic regression considering as reference Group A tumors (capsular and ≥ 5 mm)  Multivariate models excluding variables that might be considered co-dependent:  a: without ATA, stage and BRAF, but with pathological diagnosis, adjusted for pertinent variables (*BRAF* V600E, lymph node metastasis, other thyroid neoplasms, nodular hyperplasia)  b: without ATA, stage, pathological diagnosis but with NIFTP subtype, adjusted for pertinent variables (*BRAF* V600E, other thyroid neoplasms, nodular hyperplasia)  c: without ATA, stage, pathological diagnosis, but with PMiT subtype, adjusted for pertinent variables (*BRAF* V600E, lymph node metastasis, other thyroid neoplasms, nodular hyperplasia)  d: without ATA, stage and pathological diagnosis  e: with ATA, without stage and pathological diagnosis, adjusted for pertinent variables (*BRAF* V600E, other thyroid neoplasms, nodular hyperplasia) | | | | | | | | | | | | |
