# Supplementary material for: Does the Site of Origin of the Microcarcinoma with Respect to the Thyroid Surface Matter? A Multicenter Pathologic and Clinical Study for Risk Stratification
Source: Cancers (Basel). 2020 Jan 19;12(1):246. doi: 10.3390/cancers12010246 (PMC7016743; doi:10.3390/cancers12010246)
Supplement: Supplementary file 1 [file cancers-12-00246-s001.zip › 4.cancers-683690-Supplementary Material.docx]

SUPPLEMENTARY MATERIAL

Supplemental Materials and Methods

*Case selection*

Papillary microcarcinomas included in the study were previously analysed as part of a multi-institutional Italian study [1]. They were contributed from six Italian medical institutions covering different geographical areas: Bellaria and Maggiore Hospitals in Bologna (Northern Italy); University Hospital-Università “Sapienza” in Rome (Central Italy); “Casa Sollievo della Sofferenza” Hospital in San Giovanni Rotondo, and the city hospitals of Matera and Catanzaro (Southern Italy).

For selection all cases were subjected to central review. This included microscopic review of histology sections by two pathologists with a special interest in thyroid pathology (GT and ADL) to analyze tumor diagnosis, tumor subtype, tumor stage and all the variables listed below. All pathology reports and pertinent data from the patient charts were also reviewed to define the clinical and pathologic variables listed below.According to current criteria, we considered microcarcinoma a papillary carcinoma with a size smaller or equal to one centimeter, regardless of whether the tumor was discovered incidentally or following a targeted fine needle aspiration procedure [2,3]. All microcarcinomas were subtyped according to criteria used for tumors larger than one centimeter [2]

Inclusion criteria were the same as those previously reported [1]. Briefly, inclusion criteria included: a diagnosis of microcarcinoma (see above), availability of relevant clinicopathologic data, availability of Hematoxylin and Eosin (H&E) stained histology slides for review of the case, adequate material for *BRAF* V600E mutation analysis; cases where the microcarcinoma co-existed with a papillary carcinoma larger than one centimeter were excluded.

*Location of the microcarcinoma within the thyroid*

The estimated measurement of the distance of center of the tumor from the thyroid capsule was used to assess its capsular vs. subcapsular location. The distance of the center of the tumor from the thyroid capsule was calculated as follows:

Distance of tumor edge from thyroid capsule (mm) + [Tumor diameter (mm)/2]; both the distance of the tumor edge from the thyroid capsule (i.e. the distance between the edge of the tumor closest to the thyroid capsule and the thyroid capsule itself) and the tumor diameter were carefully measured micrometrically on the H&E histology sections, after review of the pathology report.

The thyroid capsule is the thin fibrous layer that separates the thyroid gland from the surrounding tissues [4]. It was identified on the histology sections as the smooth regular edge at the surface of the thyroid parenchyma. According to the above algorithm, the distance of the center of a tumor from the thyroid capsule with extrathyroidal extension (even if microscopic) is inferior to its radius.

*Clinical and pathologic data*

Pathologic and clinical features of the tumors were analized and recorded in a database, as previously reported [1].

Clinical data

Variables reported in Table 1, Table 2, Table S 1, and Tables S2a and S2b. Clinical information, including demographical data (date of birth, sex), age at the time of the diagnosis, date of surgery, staging information, administration of radioactive iodine (RAI) (with date of treatment and doses, when the information was available) were obtained by the review of the medical records. The diagnosis of the microcarcinoma was considered incidental if the tumor was not suspected before surgery, based on clinical judgment, imaging studies and the results of fine needle aspiration biopsy. The date of surgery was counted as the date of diagnosis and the beginning of the follow-up period. Stage at diagnosis was defined according to current criteria from the American Joint Committee on Cancer (AJCC) [5]. Follow-up information, as documented in the patient chart following conventional standards by physical exam, thyroglobulin measurements, as well as imaging and scintigraphic studies, was recorded in a database. Lymph node involvement was scored as pN0 or pN1 after review of the pathology report and confirmation by microscopic exam of the pertinent H&E slides; at surgery lymph nodes were sampled if there was a clinical suspicion of nodal metastases, and cases without lymph node excision were considered pN0; for pN1 cases the anatomic distribution of metastatic spread was recorded as limited to central compartment (level VI) lymph nodes, limited to lateral cervical lymph nodes homolateral to the largest tumor; limited to lateral cervical lymph nodes contralateral to the largest tumor, involvement of central compartment lymph nodes and lateral cervical lymph nodes homolateral to the largest tumor, involvement of central compartment lymph nodes and lateral cervical lymph nodes contralateral to the largest tumor, bilateral involvement of lateral cervical lymph nodes (regardless of whether lymph nodes of the central compartment were involved).

After review of the microscopic findings and of clinical data, patients were retrospectively assigned to a low risk or an intermediate risk of recurrence according to the 2015 guidelines of the American Thyroid Association (consistent with the patient population and the nature of the study that is based on microcarcinomas no patient fit the criteria for assignment to a high risk category) [6].

Disease status was classified as follows: “excellent response” when there was no biochemical (abnormal thyroglobulin values), structural (imaging studies) or clinical evidence of disease; “structural incomplete response”, when imaging studies indicated evidence of disease. Abnormal thyroglobulin values alone (“biochemical incomplete response”) were not considered as evidence of disease [7].

The presence of pathology additional to the mPTC (Hashimoto thyroiditis, nodular hyperplasia, neoplasms other than papillary carcinoma) was defined by chart review and verified histologically on all cases.

Unfavorable tumor-related patient events were classified as persistent disease if there was evidence of disease within one year of the initial diagnosis, and recurrent disease if disease was documented after one year of the diagnosis; the status of the patient at the date of the last follow-up was also recorded.

The presence and type of thyroid disease co-existing with the microcarcinoma were documented by the histologic review of the H&E slides and recorded.

Tumor subtype

Variables reported in Table 1, Table 2, Table S1, and Tables S2a and S2b. The subtype of the tumor was defined according to standard criteria used for tumors larger than one centimeter [2], with the exception of the tall cell variant of papillary carcinoma that was defined as a tumor composed for more than 50% by cells displaying tall cell features (i.e. a height at least twice their width, fully developed nuclear features of papillary carcinoma and an intensely eosinophilic cytoplasm) [2].

The categories of NIFTP (Noninvasive follicular thyroid neoplasm with papillary-like nuclear features) [8,9] and of PMiT (Papillary microtumor) [10] were also applied according to the published diagnostic criteria.

Characteristics of tumor growth

Intraglandular tumor spread and number of tumor foci: we recorded the occurrence and extent of intraglandular tumor spread; for the purpose of the study intraglandular tumor spread is defined as the presence of neoplastic cell aggregates separated from a principal tumor mass by at least one layer of non-neoplastic thyroid; the amount of intraglandular tumor spread was subdivided into absent (no intraglandular tumor spread foci), small (3 or less foci), large (>3 foci); the finding of neoplastic cells in vascular channels that could be clearly identified as such on the H&E slides was recorded as vascular invasion (not as intraglandular tumor spread); for the purpose of the study tumor multicentricity is defined as the presence in the H&E sections of two (or more) separate tumor foci of similar dimensions (at least 50% of the largest tumor diameter as measured on the H&E slide, irrespective of the histologic appearance of the tumor), or the presence of at least two separate tumors of different morphology, regardless of their diameter measured on the H&E slide (viceversa, the presence in the H&E sections of multiple separate tumor foci of different dimensions - less than 50% of the largest tumor diameter as measured on the H&E slide - and with the same morphologic appearance is defined as multifocality due to intraglandular tumor spread); we recorded the occurrence of multicentricity as defined above and the number of the multicentric microcarcinoma foci in each thyroidectomy specimen; since according to the above definition multicentric microcarcinomas may represent synchronous independent tumors, when multicentric microcarcinoma foci could be separately analysed for *BRAF* mutation their morphologic features were separately recorded in the database, and each multicentric microcarcinoma was evaluated and subjected to statistical analysis as an individual case. Additional characteristics recorded after microscopic evaluation of the H&E sections are listed below. The presence of a defined tumor capsule and its infiltration by neoplastic cells when present. The features of the border between the tumor and the surrounding non-neoplastic tissue (irregular/infiltrative or pushing/smooth). The presence of a cystic component. Whether the microcarcinoma developed within a larger hyperplastic nodule. Presence and degree of vascular invasion (i.e. the presence of neoplastic cells within vascular spaces that could be unequivocally identified as such on the H&E slides). Presence of foci of necrosis within the tumor. Presence of mitoses after the examination of 20 high power fields (X400) with an Olympus BX51 microscope, corresponding to an area of 4.8 mm2. The presence of mitoses and/or necrosis was annotated “high-grade features” in the database. Presence and extent of invasion of extrathyroidal tissues: one submillimetric focus of neoplastic cells growing in the extrathyroidal soft tissues was scored as “minimal” extrathyroidal extension, the presence of more than one such focus or the finding of a single focus > or = to one millimeter was scored as “conspicuous” extrathyroidal extension). Status of the surgical margins: negative, focally positive (focus of neoplastic cells at the margin measuring less than two millimeters), extensively positive (focus of neoplastic cells at the margin equal to or greater than two millimeters). Fibrosis associated with the tumor: absent, mild (few inconspicuous or delicate fibrous areas), moderate/extensive (clearly recognizable with multiple desmoplastic fibrotic bands within and at the periphery of the tumor nodule). Presence and amount of psammoma bodies in the surrounding non-neoplastic tissue: absent 0, 1-5-few, >5-many. Presence and amount of lymphoid cell infiltration inside the tumor and at its interface with the surrounding non-neoplastic tissue.

Microscopic appearance of the tumor

Variables reported in Table 1, Table 2, Table S1, Tables S2a and S2b. The following characteristics were studied after the examination of at least 10 high power fields (X400). Nuclear pseudoinclusions: absent (0 neoplastic cells), few (present in 1-3 neoplastic cells), many (present in >3 neoplastic cells); nuclear grooves: few (present in 0-20% neoplastic cells), many (present in 20-60% neoplastic cells), widespread (present in >60% neoplastic cells); nuclear membrane irregularities (other than nuclear grooves): few (present in 0-20% neoplastic cells), many (present in 20-60% neoplastic cells), widespread (present in >60% neoplastic cells); enlarged nuclei with complete clearing of nuclear chromatin (optically clear “Little Orphan Annie eye” nuclei): few (present in 0-20% neoplastic cells), many (present in 20-60% neoplastic cells), widespread (present in >60% neoplastic cells); neoplastic cells with intensely eosinophilic cytoplasm: few (present in 0-20% neoplastic cells), many (present in 20-60% neoplastic cells), widespread (present in >60% neoplastic cells); neoplastic cells with cytoplasmic clearing: few (present in 0-20% neoplastic cells), many (present in 20-60% neoplastic cells), widespread (present in >60% neoplastic cells); tall cells (as defined above): absent (0 neoplastic cells), few (present in 1-50% neoplastic cells), many (present in >50% neoplastic cells), the percentage of tall cells was estimated and recorded for each tumor; presence and amount of psammoma bodies within the tumor: absent (0 psammoma bodies), few (1-5 psammoma bodies), many (>5 psammoma bodies); growth patterns: the percentage of papillary, follicular and solid/trabecular patterns was estimated and recorded for each tumor.

*Immunohistochemistry*

Immunohistochemistry was performed on routinely fixed, paraffin-embedded tissue sections, according to previously published protocols [11] with a Ventana Benchmark XT autostainer (Ventana Medical Systems, Tucson, AZ, USA) using Ventana antibodies to cytokeratin 19 (papillary carcinoma marker; Clone A53-B/A2.26, Prediluted Cell Marque, Rocklin, CA, U.S.A.), CD31 (expressed by endothelial cells of blood and lymphatic vessels; Clone JC70, Prediluted Cell Marque, Rocklin, CA, U.S.A.), CD34 (expressed by endothelial cells of blood and lymphatic vessels; Clone QBEnd/10, Prediluted Ventana Benchmark, Ventana Medical Systems, Tucson, AZ, U.S.A.), the mouse monoclonal antibody to Popodoplanin (marker specific for lymphatic endothelial cells; Clone D2-40, Prediluted Cell Marque, Rocklin, CA, U.S.A.), a BRAF V600E-specific antibody (clone VE1, Ventana Medical Systems, Tucson, AZ, U.S.A.).

*BRAF V600E mutation analysis*

Histology slides were reviewed to identify the paraffin blocks with microcarcinoma. Two to six 10 µm thick sections of formalin fixed-paraffin-embedded (FFPE) material were cut from blocks with tumor, followed by one H&E control slide. The tumor area selected for the analysis was marked on the control slide, and tumor material was manually dissected under microscopic guidance from the corresponding 10 µm sections using a sterile blade. Dissected tumor areas ranged from 1 mm2 to 1.0 cm2, depending on the dimensions of the microcarcinoma. DNA was extracted from FFPE using the High Pure PCR Template Preparation Kit (Roche Diagnostic, Mannheim, Germany), according to the manufacturer's recommendation. The *BRAF* V600E mutation was analysed using Allele Specific Locked Nucleic Acid PCR (ASLNAqPCR), a mutation specific real-time PCR method that utilizes locked nucleic acid (LNA)-modified allele specific primers and an LNA-modified beacon probe to achieve high sensitivity and specificity in the identification of “hot spot” mutations [12]. An average of 20 ng of DNA were PCR amplified in separate real time reactions with allele specific primers for both wild-type and V600E mutated *BRAF*, using an ABI SDS 7000TM instrument (Applied Biosystems, Foster City, CA), and following protocols previously validated to reliably identify the *BRAF* V600E mutation in FFPE material [12]. Non mutated *BRAF* wild-type controls were analysed in parallel with each run of samples.

**Supplemental Results**

*Relationship between the distance of the center of the tumor from the thyroid surface and clinicopathologic variables*

The relationship between the distance of the center of the tumor from the thyroid surface and clinicopathologic variables - including tumor subtype and *BRAF* V600E status - the characteristics of tumor growth and the microscopic appearance of the tumor are summarized in Table S1. Univariate analysis highlights a significant correlation with important characteristics of the mPTCs. Among clinicopathologic variables - as the distance of the tumor center from the capsule increases - mPTCs tend to belong to the tall cell or classic papillary carcinoma histotype (P = 0.0003), to harbor *BRAF* V600E mutation, to have a higher probability of recurrence (P = 0.015), and to lack an association with additional thyroid neoplasms within the gland (P = 0.0037). Important features of tumor growth are also associated with the location of the center of the tumor. As the distance of the tumor center increases, there is a higher proportion of cases with high-grade features (P = 0.002), mitoses (P = 0.0056), vascular invasion (P = 0.004), intraglandular tumor spread (P < 0.0001), psammoma bodies in the parenchyma surrounding the mPTC (P = 0.004), both intra- and peritumoral lymphoid cells (P = 0.005 and P = 0.03, respectively), a cystic component (P = 0.009). Among the microscopic features, the following are more common in mPTC located further away from the thyroid capsule: pseudoinclusions (P = 0.027), grooves (P = 0.023), nuclear membrane irregularities (P = 0.0058), cytoplasmic eosinophilia (P = 0.003), cytoplasmic clearing (P = 0.039), tall cells features (P = 0.0025), psammoma bodies within the mPTC (P = 0.0039) and papillary vs. follicular pattern of growth (P = 0.004). Taken together, these data seem to indicate that tumors with aggressive features arise further away from the thyroid capsular surface, but the data are

heavily biased by the tumor size.

References

1. Tallini, G.; de Biase, D.; Durante, C.; Acquaviva, G.; Bisceglia, M.; Bruno, R.; Bacchi Reggiani, M.L.; Casadei, G.P.; Costante, G.; Cremonini, N., et al. *BRAF* V600E and risk stratification of thyroid microcarcinoma: a multicenter pathological and clinical study. *Mod Pathol* **2015**, *28*, 1343-1359, doi:10.1038/modpathol.2015.92.

2. Lloyd, R.V.; Osamura, R.Y.; Klöppel, G.; Rosai, J. WHO Classification of Tumours of Endocrine Organs. 4th ed.; IARC Press: Lyon (France), 2017; Vol. 10.

3. Tallini, G.a.G.T. Thyroid gland. In *Rosai and Ackerman's Surgical Pathology*, 11th ed.; Goldblum, L., McKenney and Myers, Ed. Elsevier: 2018; Vol. 2, pp. 302-303.

4. Rosai, J.; DeLellis, R.A.; Carcangiu, M.L.; Frabel, W.J.; Tallini, G. *Tumors of the Thyroid and Parathyroid Glands.*; Pathology, A.R.o., Ed. Silver Spring: Maryland USA, 2014; Vol. Fascicle 21.

5. Amin, M.B.; Edge, S.; Greene, F.; Byrd, D.R.; Brookland, R.K.; Washington, M.K.; Gershenwald, J.E.; Compton, C.C.; Hess, K.R. *AJCC Cancer Staging Manual*8th ed.; Springer International Publishing:  American Joint Commission on Cancer: 2017.

6. Haugen, B.R.; Alexander, E.K.; Bible, K.C.; Doherty, G.M.; Mandel, S.J.; Nikiforov, Y.E.; Pacini, F.; Randolph, G.W.; Sawka, A.M.; Schlumberger, M., et al. 2015 American Thyroid Association Management Guidelines for Adult Patients with Thyroid Nodules and Differentiated Thyroid Cancer: The American Thyroid Association Guidelines Task Force on Thyroid Nodules and Differentiated Thyroid Cancer. *Thyroid* **2016**, *26*, 1-133, doi:10.1089/thy.2015.0020.

7. Tuttle, R.M.; Tala, H.; Shah, J.; Leboeuf, R.; Ghossein, R.; Gonen, M.; Brokhin, M.; Omry, G.; Fagin, J.A.; Shaha, A. Estimating risk of recurrence in differentiated thyroid cancer after total thyroidectomy and radioactive iodine remnant ablation: using response to therapy variables to modify the initial risk estimates predicted by the new American Thyroid Association staging system. *Thyroid* **2010**, *20*, 1341-1349, doi:10.1089/thy.2010.0178.

8. Nikiforov, Y.E.; Seethala, R.R.; Tallini, G.; Baloch, Z.W.; Basolo, F.; Thompson, L.D.; Barletta, J.A.; Wenig, B.M.; Al Ghuzlan, A.; Kakudo, K., et al. Nomenclature Revision for Encapsulated Follicular Variant of Papillary Thyroid Carcinoma: A Paradigm Shift to Reduce Overtreatment of Indolent Tumors. *JAMA Oncol* **2016**, *2*, 1023-1029, doi:10.1001/jamaoncol.2016.0386.

9. Xu, B.; Farhat, N.; Barletta, J.A.; Hung, Y.P.; Biase, D.; Casadei, G.P.; Onenerk, A.M.; Tuttle, R.M.; Roman, B.R.; Katabi, N., et al. Should subcentimeter non-invasive encapsulated, follicular variant of papillary thyroid carcinoma be included in the noninvasive follicular thyroid neoplasm with papillary-like nuclear features category? *Endocrine* **2018**, *59*, 143-150, doi:10.1007/s12020-017-1484-1.

10. Rosai, J.; LiVolsi, V.A.; Sobrinho-Simoes, M.; Williams, E.D. Renaming papillary microcarcinoma of the thyroid gland: the Porto proposal. *Int J Surg Pathol* **2003**, *11*, 249-251, doi:10.1177/106689690301100401.

11. Durante, C.; Tallini, G.; Puxeddu, E.; Sponziello, M.; Moretti, S.; Ligorio, C.; Cavaliere, A.; Rhoden, K.J.; Verrienti, A.; Maranghi, M., et al. BRAF(V600E) mutation and expression of proangiogenic molecular markers in papillary thyroid carcinomas. *Eur J Endocrinol* **2011**, *165*, 455-463, doi:10.1530/EJE-11-0283.

12. Morandi, L.; de Biase, D.; Visani, M.; Cesari, V.; De Maglio, G.; Pizzolitto, S.; Pession, A.; Tallini, G. Allele specific locked nucleic acid quantitative PCR (ASLNAqPCR): an accurate and cost-effective assay to diagnose and quantify KRAS and *BRAF* mutation. *PLoS One* **2012**, *7*, e36084, doi:10.1371/journal.pone.0036084.
